# Supplementary material for: The evolution of substrate discrimination in macrolide antibiotic resistance enzymes
Source: Nat Commun. 2018 Jan 9;9:112. doi: 10.1038/s41467-017-02680-0 (PMC5760710; doi:10.1038/s41467-017-02680-0)
Supplement: Supplementary file 1 — Supplementary Information [file 41467_2017_2680_MOESM1_ESM.pdf]

Supplementary Figures

	MphA	MphB	MphC	MphE	MphF	MphG	MphH	MphI	MphJ	MphK	MphL	MphM	MphN
MphA													
MphB	37												
MphC	33	51											
MphE	34	33	31										
MphF	40	33	34	35									
MphG	38	39	36	58	38								
MphH	40	35	30	31	42	36							
MphI	38	43	41	35	36	39	39						
MphJ	39	42	41	36	37	38	37	51					
MphK	37	42	40	35	34	38	37	54	46				
MphL	34	45	40	32	34	34	30	36	39	35			
MphM	34	67	50	32	36	40	30	42	43	42	44		
MphN	33	43	41	33	35	35	30	36	39	38	65	45	
MphO	40	34	29	31	39	37	71	36	34	35	32	31	30

Supplementary Figure 1: Pair-wise sequence relationship of Mphs. Values are coloured from lowest (white) to highest (red), and the value is the pair-wise protein identity.

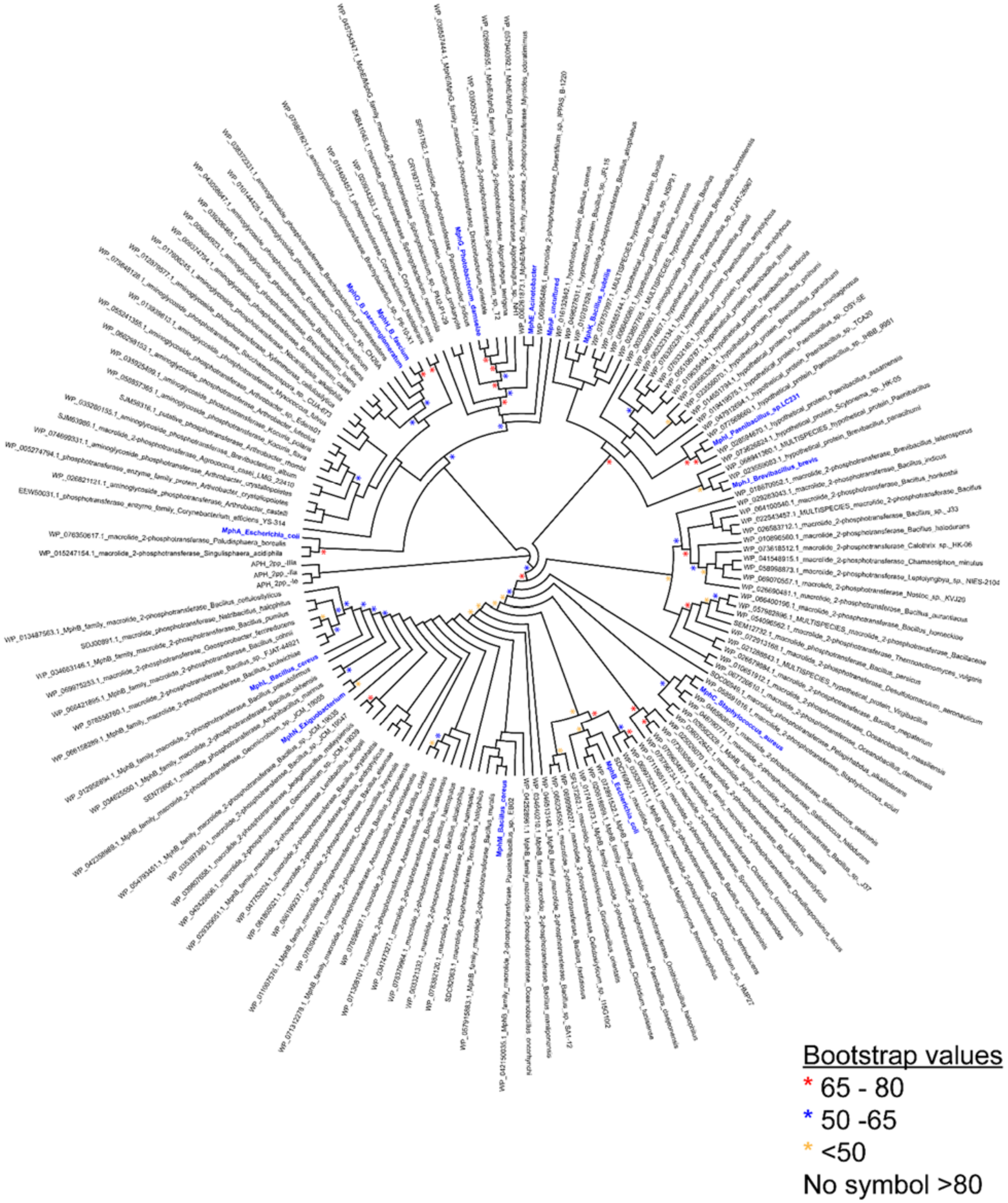

Supplementary Figure 2: Cladogram of the Mph family. This figure is identical to Figure 3, but with fully labelled leaves and bootstrap values. Bootstrap values above 80 are not shown for presentation.

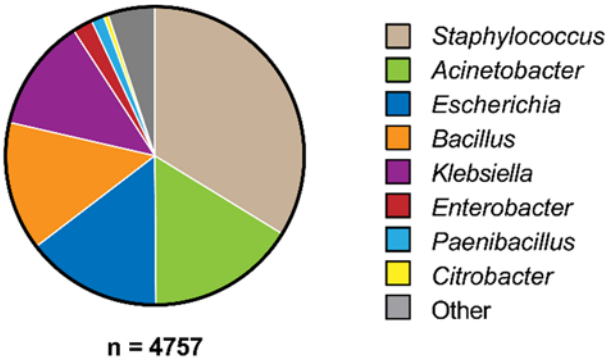

Supplementary Figure 3: Taxonomic distribution of all Mphs detected in RefSeq genomes.

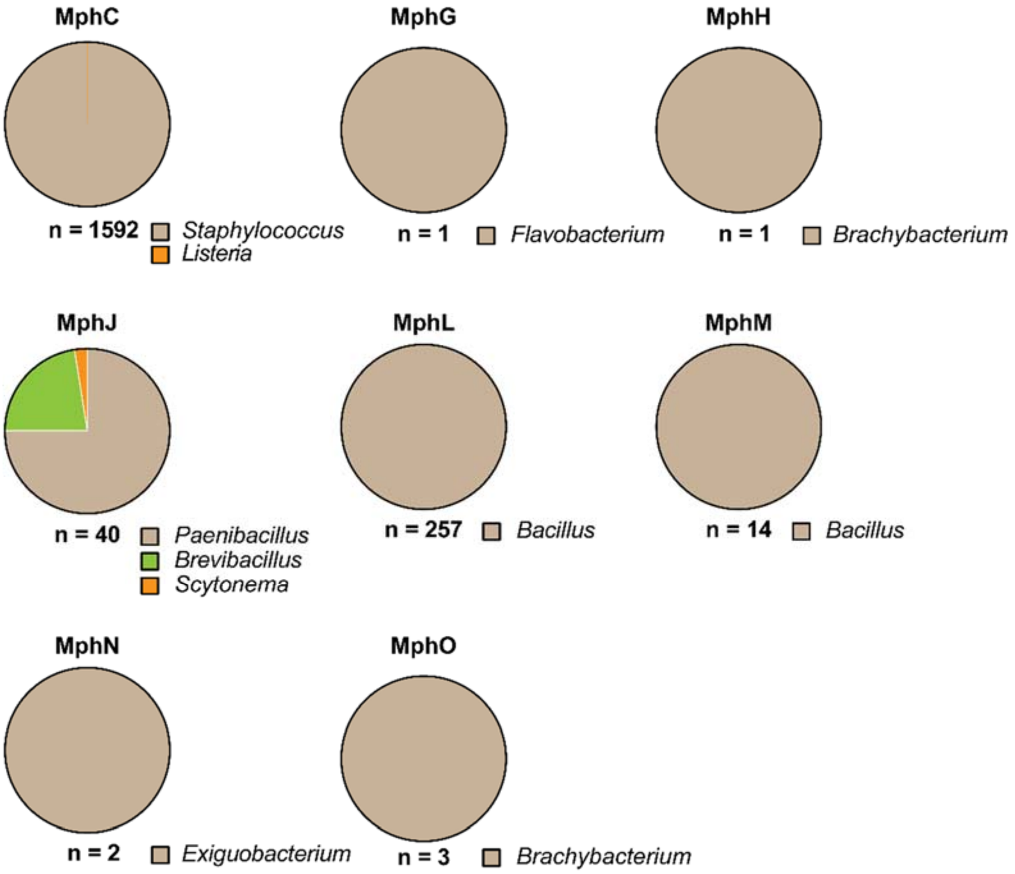

Supplementary Figure 4: Taxonomic distribution of Mph homologs within 80% sequence identity.

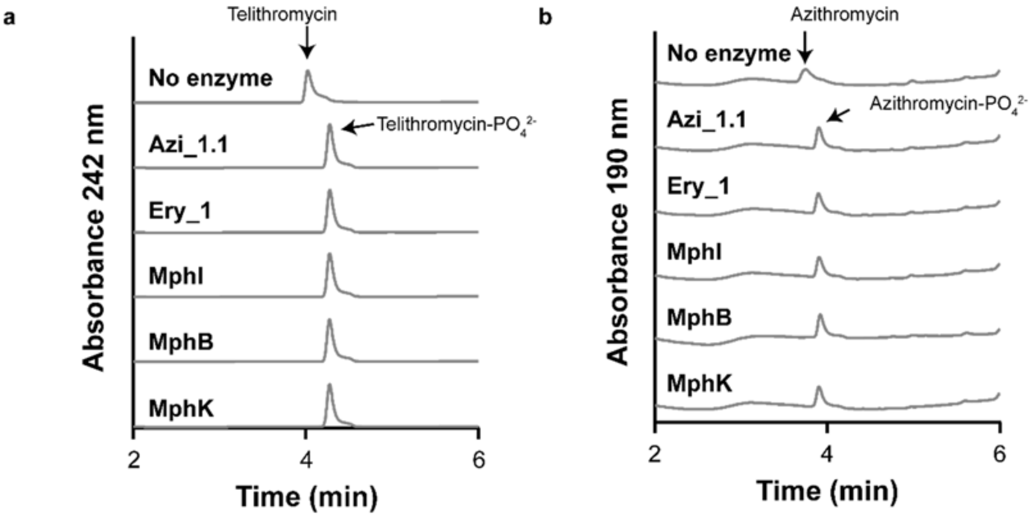

Supplementary Figure 5: Functional validation of Mphs. Enzyme reactions were analyzed with HPLC and confirmed to modify azithromycin (**a**) and telithromycin (**b**).

a

b

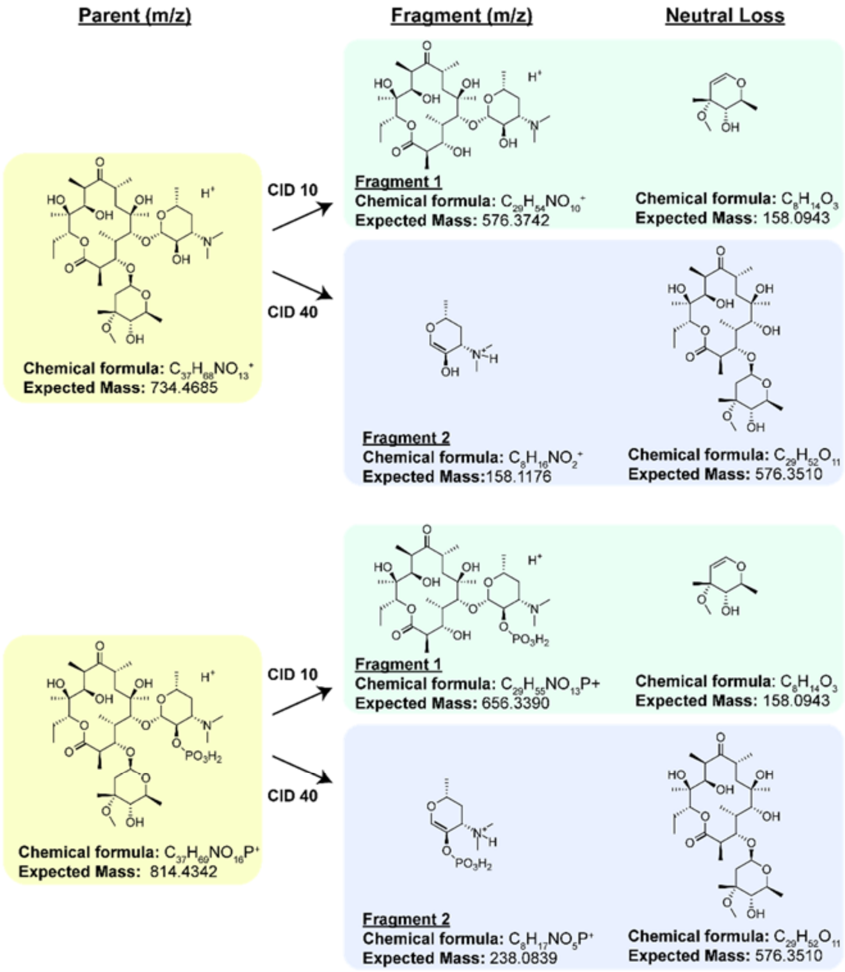

c

Enzyme	Fragment	*m/z* [M+H]+ Substrate	*m/z* [M+H]+ Product	Mass difference
MphB	Parent	734.4687	814.4337	79.9650
	Frag. 1	576.3730	656.3390	79.9660
	Frag. 2	158.1174	238.0833	79.9659
MphI	Parent	734.4687	814.4344	79.9657
	Frag. 1	576.3730	656.3395	79.9665
	Frag. 2	158.1174	238.0831	79.9657
MphK	Parent	734.4687	814.4343	79.9656
	Frag. 1	576.3730	656.3391	79.9661
	Frag. 2	158.1174	238.0830	79.9656
Azi_1.1	Parent	734.4687	814.4340	79.9653
	Frag. 1	576.3730	656.3390	79.9660
	Frag. 2	158.1174	238.0834	79.9660
Ery_1	Parent	734.4687	814.4336	79.9649
	Frag. 1	576.3730	656.3388	79.9658
	Frag. 2	158.1174	238.0833	79.9659

Supplementary Figure 6: Structural characterization of phosphorylated macrolides. Fragmentation patterns of (**a**) erythromycin and (**b**) phosphorylated erythromycin. Charged fragments and neutral losses, and exact masses are shown. (**c**) Mass fragments observed using tandem mass spectrometry.

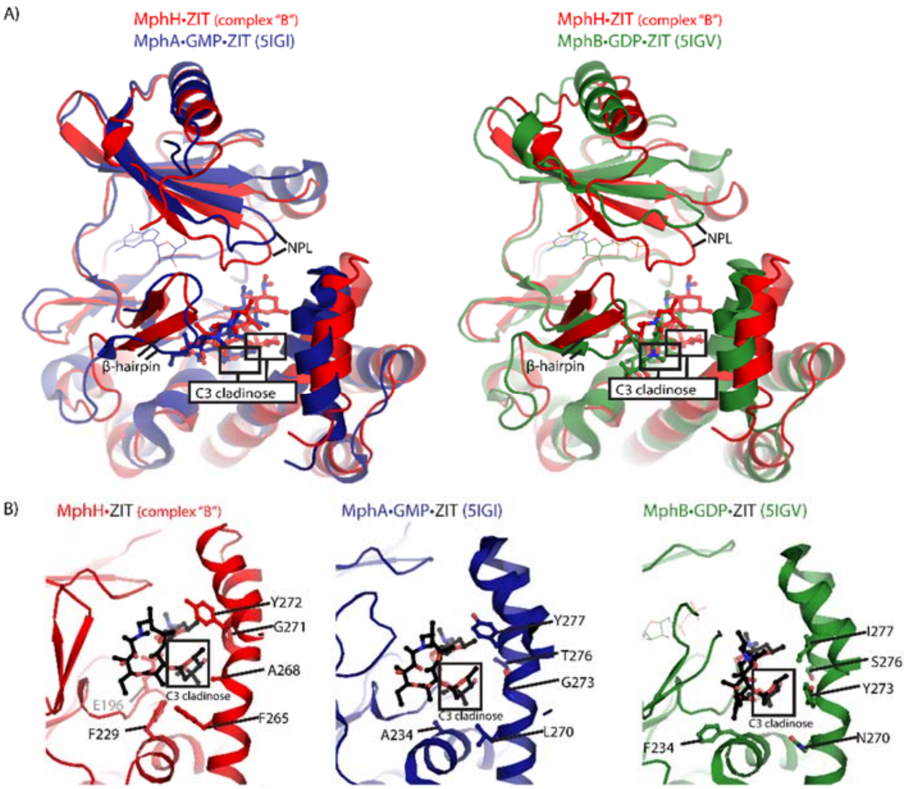

Supplementary Figure 7: (**a**) Crystal structure of MphB apoenzyme. (**b**) Superposition of crystal structures of MphB and MphH•azithromycin (complex "A"). (**c**) Electrostatic surface representations of MphB, MphH•azithromycin (complex "A") and MphH•azithromycin (complex "B").

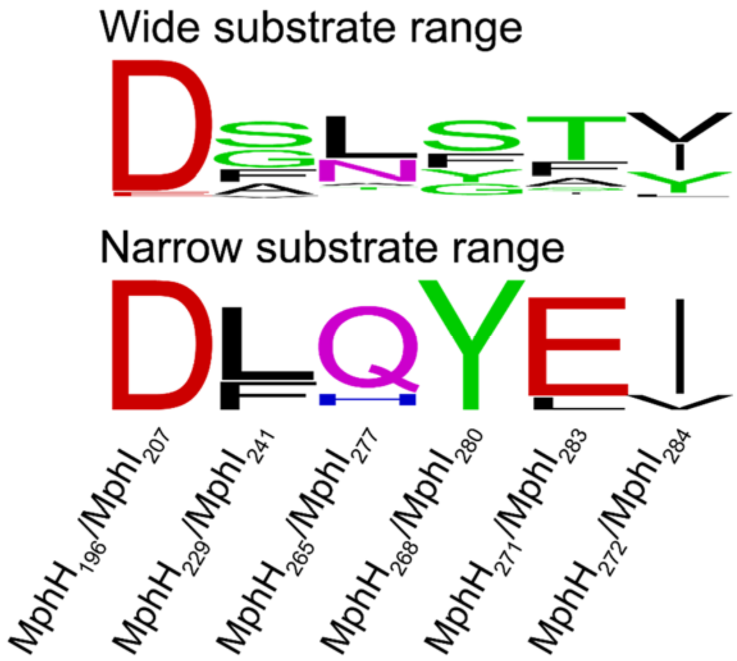

Supplementary Figure 8: Conservation of residues that contact the cladinose in the MphH crystal structure with azithromycin bound. Sequence conservation is displayed as a sequence logo created with WebLogo. Wide substrate range includes sequences in all clades except MphI and MphK; narrow substrate range includes sequences in only MphI and MphK clades.

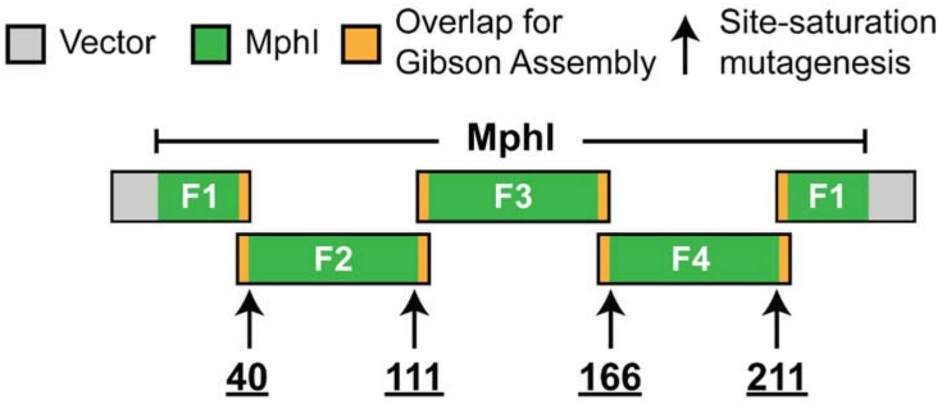

Supplementary Figure 9: Overview of the site-saturation combinatorial mutagenesis approach used to generate the MphI mutant library.

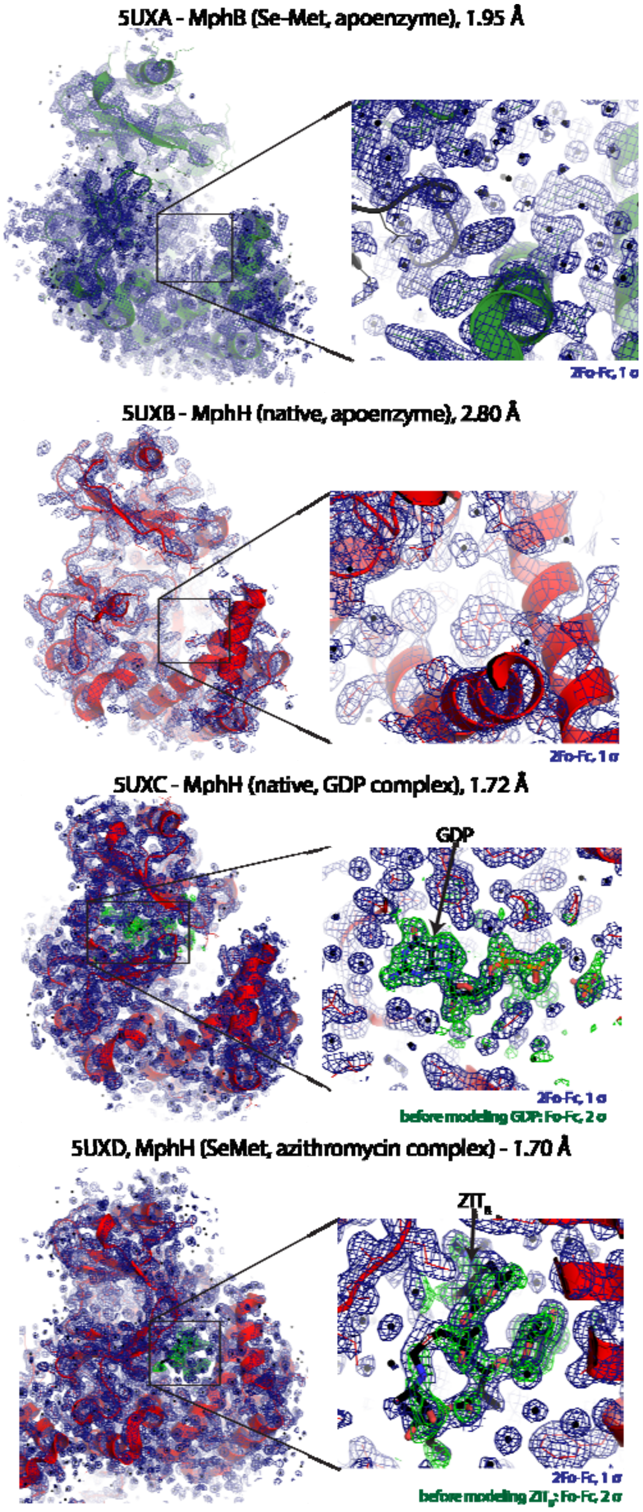

Supplementary Figure 10: Electron density map of crystal structures in this study.

Supplementary Tables

Supplementary Table 1: Macrolide phosphotransferases used in this study and their revised nomenclature.

Name	Previous name	Organism	Accession	Reference	Location
MphA		*Escherichia coli*	WP_023063803.1	[1]	Mobile element
MphB		*Escherichia coli*	WP_000031017.1	[2]	Mobile element
MphC		*Staphylococcus aureus*	BAD86538.1	[3]	Mobile element
MphE		uncultured	ABI20451.1	[4]	Mobile element
MphF[b]	MphE	uncultured	CAJ98570.1	[5]	Mobile element
MphG		*Photobacterium damselae*	BAL43359.1	[6]	Mobile element
MphH[c]	MphE	*Brachybacterium faecium*	WP_015776248.1	[7]	Chromosome
MphI		*Paenibacillus* sp. LC231	KX531056.1	[8]	Chromosome
MphJ		*Brevibacillus brevis* VM4	KY753883	Submitted	Chromosome
MphK	YcbJ[d]	*Bacillus subtilis* 168	WP_003246254.1	This study	Chromosome
MphL	B. cereus Cluster A	*Bacillus cereus*	WP_001094257.1	[9]	Chromosome
MphM	B. cereus Cluster B	*Bacillus cereus*	WP_001041372.1	[9]	Mobile element[a]
MphN	mph_like	*Exiguobacterium* sp. S3-2	AHE40505.1	[10]	Mobile element
MphO[c]	MphE	*Brachybacterium paraconglomeratum*	WP_050815728.1	[7]	Chromosome

[a]The upstream and downstream region of MphM was similar to *Bacillus thurengiensis* plasmids, but was not conclusively shown to be on a plasmid.
[b]MphF was originally annotated MphE, but renamed in ref. [11] because MphE already existed.
[c]MphH and MphO were both originally named MphE in ref. [7], but were renamed in this study because MphE already existed. Furthermore, according to the macrolide resistance nomenclature proposed in ref. [12], MphH and MphO were assigned different names because they share <80% identity.
[d]MphK was annotated as YcbJ in standard *Bacillus subtilis* gene nomenclature[13].

Supplementary Table 2: Antibiotic susceptibility of *mph*s expressed in *E. coli* TOP10.

Antibiotic	Empty vector	***mphB***	***mphK***	***mphH***	***mphI***
Erythromycin	64	256	64	64 - 128	64
Clarithromycin	64	>128	64	128	64
Azithromycin	4	16	4	4	4
Telithromycin	8	512	256	16	512
Spiramycin	256	1024	512	256	1024
Tylosin	512	>2048	2048	1024	>2048
Kanamycin	0.5	0.5	0.5	0.5	0.5

Supplementary Table 3: Mass characterization of macrolide antibiotics modified by Mphs.

Resistance enzyme	Substrate	*m/z* (substrate)	*m/z* (product)	Mass difference
MphB	Azithromycin	749.2	829.1	79.9
	Telithromycin	812.3	892.3	80.0
MphI	Azithromycin	749.2	829.1	79.9
	Telithromycin	812.3	892.2	79.9
MphK	Azithromycin	749.2	829.2	80.0
	Telithromycin	812.3	892.2	79.9
Azi_1.1	Azithromycin	749.2	829.1	79.9
	Telithromycin	812.3	892.3	80.0
Ery_1	Azithromycin	749.2	829.2	80.0
	Telithromycin	812.3	892.3	80.0

Supplementary Table 4: X-ray diffraction data collection and refinement statistics

Structure	MphB (Se-Met, apoenzyme)	MphB (native, apoenzyme)	MphH (native)•GDP+P_i complex	MphH (Se-Met)•azithromycin complex
PDB Code	5UXA	5UXB	5UXC	5UXD
**Data collection**				
Space group	$C222_1$	$P2_12_12_1$	$P2_12_12_1$	$P\ 2_1$
Cell dimensions (*a*, *b*, *c* Å; β °)	67.28, 116.68, 92.58; 90	75.93, 79.74, 99.13; 90	49.56, 77.00, 94.41; 90	55.05, 81.21, 71.34; 96.83
Resolution, Å	30.00 – 1.95	30.00 – 2.80	43.88 – 1.72	70.83 – 1.70
R_{merge}[a]	0.092 (0.793)[b]	0.143 (0.593)	0.086 (0.588)	0.086 (0.845)
R_{pim}	0.033 (0.282)	0.048 (0.200)	0.069 (0.480)	0.043 (0.417)
$CC_{1/2}$	(0.905)[b]	(0.962)	(0.994)	(0.706)
I / σ(I)	24.9 (3.5)	16.3 (3.8)	9.1 (2.4)	10.4 (1.6)
Completeness, %	100 (100)	99.9 (100)	99.9 (100)	98.1 (100)
Redundancy	8.6 (8.7)	9.6 (9.5)	4.0 (4.0)	4.9 (5.0)
**Refinement**				
Resolution, Å	29.17 – 1.94	28.75 – 2.79	40.24 – 1.72	45.35 – 1.70
No. of unique reflections: working, test	26109, 1916	15089, 1243	39023, 1903	67224, 3938
R-factor/free *R*-factor[c]	15.1/20.1 (18.9/25.9)	21.9/25.2 (26.4/30.6)	16.4/20.7 (27.0/35.7)	19.2/23.2 (29.4/33.6)
No. of refined atoms, molecules				
Protein	2440, 1	4463, 2	2286, 1	4625, 2
Magnesium	N/A	N/A	1	N/A
GDP	N/A	N/A	28, 1	N/A
Other solvent	3	4	142	16

Structure	MphB (Se-Met, apoenzyme)	MphB (native, apoenzyme)	MphH (native)•GDP+P_i complex	MphH (Se-Met)•azithromycin complex
PDB Code	5UXA	5UXB	5UXC	5UXD
Water	461	118	486	1301
B-factors				
Protein	42.8	47.8	22.8	26.11
Magnesium	N/A	N/A	13.4	N/A
GDP	N/A	N/A	23.4	N/A
Other solvent	38.4	42.1	42.7	37.1
Water	46.3	38.9	39.6	39.5
r.m.s.d.				
Bond lengths, Å	0.016	0.003	0.015	0.006
Bond angles, °	1.156	0.565	1.497	0.940

[a]$R_{sym} = \Sigma_h \Sigma_i |I_i(h) - \langle I(h) \rangle / \Sigma_h \Sigma_i I_i(h)$, where $I_i(h)$ and $\langle I(h) \rangle$ are the *i*th and mean measurement of the intensity of reflection *h*.

[b]Figures in parentheses indicate the values for the outer shells of the data.

[c]$R = \Sigma|F_p^{obs} - F_p^{calc}|/\Sigma F_p^{obs}$, where F_p^{obs} and F_p^{calc} are the observed and calculated structure factor amplitudes, respectively.

Supplementary Table 5: Steady-state kinetics of MphI.

**Substrate**	**K_m (μM)**	**k_{cat} (s^{-1})**	**K_i (μM)**	**k_{cat}/K_m**
Erythromycin	110 ± 10	0.012 ± 0.001		(1.0 ± 0.1) × 10^2
Azithromycin	56 ± 3	0.0217 ± 0.0003		(3.9 ± 0.2) × 10^2
Roxithromycin	32 ± 4	0.0030 ± 0.0001		(9.1 ± 1.1) × 10^1
Telithromycin	3.0 ± 0.5	0.99 ± 0.03		(3.3 ± 0.5) × 10^5
Tylosin	2.5 ± 0.7	0.46 ± 0.02		(1.8 ± 0.5) × 10^5
Spiramycin	8.1 ± 1.8	1.7 ± 0.2	300 ± 90	(2.1 ± 0.5) × 10^5
Josamycin	30 ± 8	4.7 ± 0.6	640 ± 240	(1.6 ± 0.5) × 10^5
GTP	10 ± 1	1.02 ± 0.02		(1.0 ± 0.1) × 10^5

Supplementary Table 6: Steady-state kinetics of MphK.

Substrate	K_m (μM)	k_{cat} (s^{-1})	K_i (μM)	k_{cat}/K_m
Erythromycin	-	-	-	-
Azithromycin	940 ± 320	0.025 ± 0.005		$(2.6 \pm 1.1) \times 10^1$
Roxithromycin	100 ± 25	0.0050 ± 0.0004		$(4.9 \pm 1.2) \times 10^1$
Telithromycin	4.2 ± 0.45	0.056 ± 0.001		$(1.3 \pm 0.1) \times 10^4$
Tylosin	6.3 ± 2.4	0.0022 ± 0.0002		$(3.5 \pm 1.4) \times 10^2$
Spiramycin	7.9 ± 1.1	0.044 ± 0.002	750 ± 160	$(5.7 \pm 0.9) \times 10^3$
Josamycin	6.389 ± 1.938	0.097 ± 0.016	37 ± 12	$(1.5 \pm 0.5) \times 10^4$
GTP	17 ± 1	0.0491 ± 0.0004		$(3.0 \pm 0.1) \times 10^3$

Supplementary Table 7: Antibiotic susceptibility of *mph*s expressed in *E. coli* BW25113 Δ*bamB*Δ*tolC*.

Antibiotic	Empty vector	*mphB*	*mphK*	*mphH*	*mphI*
Erythromycin	0.5	64	1	16	1
Clarithromycin	0.5	32	0.5	16	1
Azithromycin	0.063	4	0.25	0.5	0.25
Telithromycin	0.125	>16	>16	4	>16
Spiramycin	2	256	256	16	256
Tylosin	2	1024	512	64	1024
Josamycin	2-4	>64	64	8	>64
Kanamycin	1	1	1	1	1

Supplementary Table 8: Steady-state kinetics of MphB.

Substrate	K_m (μM)	k_{cat} (s^{-1})	K_i (μM)	k_{cat}/K_m
Erythromycin	1.6 ± 0.2	0.0412 ± 0.0009		$(2.5 \pm 0.3) \times 10^4$
Azithromycin	2.9 ± 0.36	0.189 ± 0.004		$(6.6 \pm 0.8) \times 10^4$
Roxithromycin	1.5 ± 0.4	0.025 ± 0.001	960 ± 510	$(1.7 \pm 0.4) \times 10^4$
Telithromycin	6.6 ± 1.8	0.77 ± 0.04		$(1.2 \pm 0.3) \times 10^5$
Tylosin	1.8 ± 0.4	0.070 ± 0.005	350 ± 160	$(9.4 \pm 4.5) \times 10^4$
Spiramycin	2.0 ± 0.9	0.11 ± 0.02	39 ± 16	$(5.5 \pm 2.7) \times 10^4$
Josamycin	0.83 ± 0.27	1.2 ± 0.1	94 ± 27	$(1.5 \pm 0.5) \times 10^6$
GTP	8.3 ± 0.45	0.602 ± 0.008		$(7.3 \pm 0.4) \times 10^4$

Supplementary Table 9: Antibiotic susceptibility of MphI and single mutations identified with ancestral sequence reconstruction expressed in *E. coli* BW25113 Δ*bamB*Δ*tolC*.

Antibiotic	*mphI*	*mphI*$_{M40L}$	*mphI*$_{S111T}$	*mphI*$_{T166M}$	*mphI*$_{P211G}$
Erythromycin	1	1	1	1	1
Clarithromycin	1	1	1-2	1	1
Azithromycin	0.25	0.25	0.25-0.5	0.25-0.5	0.25
Telithromycin	>16	>16	>16	>16	>16
Spiramycin	256	256	256	256	256
Tylosin	1024	1024	1024	1024	1024
Josamycin	>64	>64	>64	>64	>64
Kanamycin	1	1	1	1	1

Supplementary Table 10: Antibiotic susceptibility of *mphI* and two library mutants with expanded resistance to C3 cladinose macrolides expressed in *E. coli* TOP10.

Antibiotic	*mphI*	*azi_1.1*[a]	*ery_1*[b]
Erythromycin	64	128	128
Clarithromycin	64	64	64
Azithromycin	4	16	16
Telithromycin	512	512	512
Spiramycin	1024	2048	2048
Tylosin	>2048	>2048	>2048
Kanamycin	0.5	0.5	0.5

a - MphI$_{M40A/S111G/T166E/P211L}$
b - MphI$_{M40A/S111R/T166E/P211L}$

Supplementary Table 11: Steady-state kinetics of the MphI mutant Azi_1.1 (MphI$_{M40A/S111G/T166E/P211L}$).

Substrate	K_m (μM)	k_{cat} (s^{-1})	k_{cat}/K_m
Erythromycin	46 ± 6	0.197 ± 0.007	$(4.3 \pm 0.6) \times 10^3$
Azithromycin	34 ± 4	0.47 ± 0.01	$(1.4 \pm 0.2) \times 10^4$
Roxithromycin	19 ± 3	0.153 ± 0.004	$(7.9 \pm 1.1) \times 10^3$
Telithromycin	2.0 ± 0.2	0.376 ± 0.007	$(1.8 \pm 0.2) \times 10^5$
Tylosin	4.0 ± 0.6	0.131 ± 0.003	$(3.2 \pm 0.5) \times 10^4$
Spiramycin	4.2 ± 0.3	0.347 ± 0.005	$(8.2 \pm 0.6) \times 10^4$
Josamycin	4.1 ± 0.7	0.174 ± 0.006	$(4.3 \pm 0.7) \times 10^4$
GTP	5 ± 0.3	0.450 ± 0.005	$(8.3 \pm 0.4) \times 10^4$

Supplementary Table 12: Steady-state kinetics of MphI mutant Ery_1 ($MphI_{M40A/S111R/T166E/P211L}$).

Substrate	K_m (μM)	k_{cat} (s^{-1})	K_i (μM)	k_{cat}/K_m
Erythromycin	75 ± 8	0.19 ± 0.01		(2.6 ± 0.3) × 10^3
Azithromycin	32 ± 2	0.56 ± 0.01		(1.8 ± 0.1) × 10^4
Roxithromycin	46 ± 3	0.145 ± 0.002		(3.2 ± 0.2) × 10^3
Telithromycin	5.6 ± 1.1	0.72 ± 0.03		(1.3 ± 0.3) × 10^5
Tylosin	5.4 ± 0.75	0.32 ± 0.01		(5.9 ± 0.8) × 10^4
Spiramycin	15 ± 5	1.3 ± 0.2	210 ± 90	(8.7 ± 3.2) × 10^4
Josamycin	20 ± 3	0.45 ± 0.02		(2.2 ± 0.4) × 10^4
GTP	5.1 ± 0.3	0.71 ± 0.01		(1.4 ± 0.8) × 10^5

Supplementary Table 13: Steady-state kinetics of $MphI_{S111R/T166E/P211L}$.

Substrate	K_m	k_{cat} (s^{-1})	K_i (μM)	k_{cat}/K_m
Erythromycin	140 ± 20	0.0114 ± 0.0004		(7.9 ± 1.1) × 10^1
Azithromycin	93 ± 5	0.0490 ± 0.0008		(5.3 ± 0.3) × 10^2
Roxithromycin	60 ± 8	0.0054 ± 0.0002		(9.0 ± 1.2) × 10^1
Telithromycin	5.0 ± 0.7	0.154 ± 0.004		(3.1 ± 0.5) × 10^4
Tylosin	4.6 ± 0.9	0.21 ± 0.01	700 ± 260	(4.5 ± 0.9) × 10^4
Spiramycin	11 ± 2.1	0.53 ± 0.04	1500 ± 600	(4.9 ± 1.0) × 10^4
Josamycin	34 ± 3	0.95 ± 0.02		(2.8 ± 0.2) × 10^4
GTP	3.0 ± 0.5	0.090 ± 0.003		(3.0 ± 0.5) × 10^4

Supplementary Table 14: Steady-state kinetics of $MphI_{M40A/S111R/T166E}$.

Substrate	K_m (μM)	k_{cat} (s^{-1})	K_i (μM)	k_{cat}/K_m
Erythromycin	79 ± 14	0.020 ± 0.001		(2.6 ± 0.6) × 10^2
Azithromycin	83 ± 6	0.094 ± 0.002		(1.1 ± 0.1) × 10^3
Roxithromycin	100 ± 30	0.0099 ± 0.0008		(9.7 ± 2.7) × 10^1
Telithromycin	5.5 ± 0.8	1.68 ± 0.05		(3.1 ± 0.4) × 10^5
Tylosin	9.4 ± 2.7	0.70 ± 0.11	67 ± 22	(7.4 ± 2.4) × 10^4
Spiramycin	6.9 ± 1.0	1.40 ± 0.04		(2.0 ± 0.3) × 10^5
Josamycin	14 ± 3	1.8 ± 0.07		(1.2 ± 0.3) × 10^5
GTP	16 ± 1	0.78 ± 0.01		(5.0 ± 0.4) × 10^4

Supplementary Table 15: Steady-state kinetics of MphI$_{S111R/T166E}$.

Substrate	K_m (µM)	k_{cat} (s^{-1})	K_i (µM)	k_{cat}/K_m
Erythromycin	157 ± 9	0.0202 ± 0.0004		(1.28 ± 0.07) × 10^2
Azithromycin	114 ± 7	0.099 ± 0.002		(8.6 ± 0.5) × 10^2
Roxithromycin	46 ± 5	0.0066 ± 0.0001		(1.4 ± 0.2) × 10^2
Telithromycin	3.1 ± 0.5	1.32 ± 0.04		(4.3 ± 0.7) × 10^5
Tylosin	12 ± 3	0.37 ± 0.04	340 ± 120	(3.0 ± 0.8) × 10^4
Spiramycin	10 ± 1	1.01 ± 0.03		(1.0 ± 0.1) × 10^5
Josamycin	34 ± 4	3.7 ± 0.1		(1.1 ± 0.1) × 10^5
GTP	13 ± 2	0.55 ± 0.02		(4.2 ± 0.5) × 10^4

Supplementary Table 16: Oligonucleotides used in this study.

Primer	Direction	Sequence (5' to 3')
**Site-directed mutagenesis**		
A40M	f	ATGAATGAATCCGGCATGGATTTCCTTGTTGGATTCGCAGAGG
	r	TCCAACAAGGAAATCCATGCCGGATTCATTCATCTCTATTCCCTC
R111S	f	GGGCAGCCTGCCGCTTCCGTCAGCATGGAAGGATATGCATGGA
	r	TCCTTCCATGCTGACGGAAGCGGCAGGCTGCCCGCTAAGCAGC
E166T	f	GAGGTTCGTGATGAGACGGCGCGAAATATGGAAGACATCAAGAGC
	r	TTCCATATTTCGCGCCGTCTCATCACGAACCTCTTGGGGACTC
L211P	f	GGCGATCTTCATCCCCCGCATATCCTGATTGATGAGCGCGTGC
	r	ATCAATCAGGATATGCGGGGGATGAAGATCGCCATGGATGAGGGC
M40L	f	ATGAATCCGGCCTGGATTTCCTTGTTGGATTCG
	r	AAGGAAATCCAGGCCGGATTCATTCATCTCTATTCCCTCC
S111T	f	GCCTGCCGCTACCGTCAGCATGGAAGGATATGC
	r	CCATGCTGACGGTAGCGGCAGGCTGCCCGCTAAGC
T166M	f	TCGTGATGAGATGGCGCGAAATATGGAAGACATCAAGAGCCG
	r	TATTTCGCGCCATCTCATCACGAACCTCTTGGGGACTC
P211G	f	CGATCTTCATCCCGGGCATATCCTGATTGATGAGCGCGTGCAG
	r	AATCAGGATATGCCCGGGATGAAGATCGCCATGGATGAGG
**Site-saturation combinatorial mutagenesis**		
Backbone	f	CATATCCTGATTGATGAGCGC
	r	GCCGGATTCATTCATCTCTATTCC
f1	f	GGGAATAGAGATGAATGAATCCGGCnnkGATTTCCTTGTTGGATTCGCAGAGG
	r	TCCATGCATATCCTTCCATGCTGACmnnAGCGGCAGGCTGCCCGCTAAGCAGC
f2	f	GTCAGCATGGAAGGATATGC
	r	CTCATCACGAACCTCTTGGGGAC
f3	f	GAGTCCCCAAGAGGTTCGTGATGAGnnkGCGCGAAATATGGAAGACATCAAGA
	r	GCACGCGCTCATCAATCAGGATATGmnnGGGATGAAGATCGCCATGGATGAGG

Supplementary Table 17: Codon optimized *mphB* sequence.

Gene	DNA sequence
mphB	ATGAGCAAAGATATTAAACAGGTGATTGAAATTGCGAAAAAACATAACCTGTTTCTGAAAGAAGAA<br>ACCATTCAGTTTAACGAAAGCGGCCTGGATTTTCAGGCGGTGTTTGCGCAGGATAACAACGGCATTG<br>ATTGGGTGCTGCGCCTGCCGCGCCGCGAAGATGTGATGCCGCGCACCAAAGTGGAAAAACAGGCG<br>CTGGATCTGGTGAACAAATATGCGATTAGCTTTCAGGCGCCGAACTGGATTATTTATACCGAAGAAC<br>TGATTGCGTATAAAAAACTGGATGGCGTGCCGGCGGGCACCATTGATCATAACATTGGCAACTATAT<br>TTGGGAAATTGATATTAACAACGTGCCGGAACTGTTTCATAAAAGCCTGGGCCGCGTGCTGGCGGA<br>ACTGCATAGCATTCCGAGCAACAAAGCGGCGGCGCTGGATCTGGTGGTGCATACCCCGGAAGAAGC<br>GCGCATGAGCATGAAACAGCGCATGGATGCGGTGCGCGCGAAATTTGGCGTGGGCGAAAACCTGT<br>GGAACCGCTGGCAGGCGTGGCTGAACGATGATGATATGTGGCCGAAAAAAACCGGCCTGATTCATG<br>GCGATGTGCATGCGGGCCATACCATGATTGATAAAGATGCGAACGTGACCGGCCTGATTGATTGGA<br>CCGAAGCGAAAGTGACCGATGTGAGCCATGATTTTATTTTTAACTATCGCGCGTTTGGCGAAGAAGG<br>CCTGGAAGCGCTGATTCTGGCGTATAAAGAAATTGGCGGCTATTATTGGCCGAAAATGAAAGAACA<br>TATTATTGAACTGAACGCGGCGTATCCGGTGAGCATTGCGGAATTTGCGCTGGTGAGCGGCATTGA<br>AGAATATGAACAGATGGCGAAAGAAGCGCTGGAAGTGCAGGGCAGCTAA

Supplementary References

1 Shakya, T. & Wright, G. D. Nucleotide selectivity of antibiotic kinases. *Antimicrob. Agents Chemother.* **54**, 1909-1913 (2010). 10.1128/aac.01570-09.

2 Noguchi, N., Katayama, J. & O'Hara, K. Cloning and nucleotide sequence of the *mphB* gene for macrolide 2'-phosphotransferase II in *Escherichia coli*. *FEMS Microbiol. Lett.* **144**, 197-202 (1996).

3 Matsuoka, M., Inoue, M., Endo, Y. & Nakajima, Y. Characteristic expression of three genes, *msr(A)*, *mph(C)* and *erm(Y)*, that confer resistance to macrolide antibiotics on *Staphylococcus aureus*. *FEMS Microbiol. Lett.* **220**, 287-293 (2003).

4 Schluter, A. *et al.* Erythromycin resistance-conferring plasmid pRSB105, isolated from a sewage treatment plant, harbors a new macrolide resistance determinant, an integron-containing Tn402-like element, and a large region of unknown function. *Appl. Environ. Microbiol.* **73**, 1952-1960 (2007). 10.1128/aem.02159-06.

5 Szczepanowski, R., Krahn, I., Bohn, N., Puhler, A. & Schluter, A. Novel macrolide resistance module carried by the IncP-1beta resistance plasmid pRSB111, isolated from a wastewater treatment plant. *Antimicrob. Agents Chemother.* **51**, 673-678 (2007). 10.1128/aac.00802-06.

6 Nonaka, L., Maruyama, F., Suzuki, S. & Masuda, M. Novel macrolide-resistance genes, *mef(C)* and *mph(G)*, carried by plasmids from *Vibrio* and *Photobacterium* isolated from sediment and seawater of a coastal aquaculture site. *Lett. Appl. Microbiol.* **61**, 1-6 (2015). 10.1111/lam.12414.

7 Bhullar, K. *et al.* Antibiotic resistance is prevalent in an isolated cave microbiome. *PLoS One* **7**, e34953 (2012). 10.1371/journal.pone.0034953.

8 Pawlowski, A. C. *et al.* A diverse intrinsic antibiotic resistome from a cave bacterium. *Nat. Commun.* **7**, 13803 (2016). 10.1038/ncomms13803.

9 Wang, C. *et al.* Functional characterization and phylogenetic analysis of acquired and intrinsic macrolide phosphotransferases in the *Bacillus cereus* group. *Environ. Microbiol.* **17**, 1560-1573 (2015). 10.1111/1462-2920.12578.

10 Yang, J. *et al.* Characterization of a multiresistant mosaic plasmid from a fish farm Sediment *Exiguobacterium* sp. isolate reveals aggregation of functional clinic-associated antibiotic resistance genes. *Appl. Environ. Microbiol.* **80**, 1482-1488 (2014). 10.1128/AEM.03257-13.

11 Roberts, M. C., Schwarz, S. & Aarts, H. J. Erratum: Acquired antibiotic resistance genes: an overview. *Front. Microbiol.* **3**, 384 (2012). 10.3389/fmicb.2012.00384.

12 Roberts, M. C. Update on macrolide–lincosamide–streptogramin, ketolide, and oxazolidinone resistance genes. *FEMS Microbiol. Lett.* **282**, 147-159 (2008). 10.1111/j.1574-6968.2008.01145.x.

13 Kunst, F. *et al.* The complete genome sequence of the gram-positive bacterium *Bacillus subtilis*. *Nature* **390**, 249-256 (1997). 10.1038/36786.
